# Supplementary material for: Kinetic and structural roles for the surface in guiding SAS-6 self-assembly to direct centriole architecture
Source: Nat Commun. 2021 Oct 26;12:6180. doi: 10.1038/s41467-021-26329-1 (PMC8548535; doi:10.1038/s41467-021-26329-1)
Supplement: Supplementary file 1 — Supplementary Informations [file 41467_2021_26329_MOESM1_ESM.pdf]

Supplementary information

Kinetic and structural roles for the surface in guiding SAS-6 self-assembly to direct centriole architecture

Niccolò Banterle[1,7], Adrian P. Nievergelt[2,7], Svenja de Buhr[3,4], Georgios N. Hatzopoulos[1], Charlène Brillard[2], Santiago Andany[2], Tania Hübscher[1], Frieda A. Sorgenfrei[4,5], Ulrich S. Schwarz[3,6], Frauke Gräter[3,4], Georg E. Fantner[2], Pierre Gönczy[1,*]

Correspondence: pierre.gonczy@epfl.ch

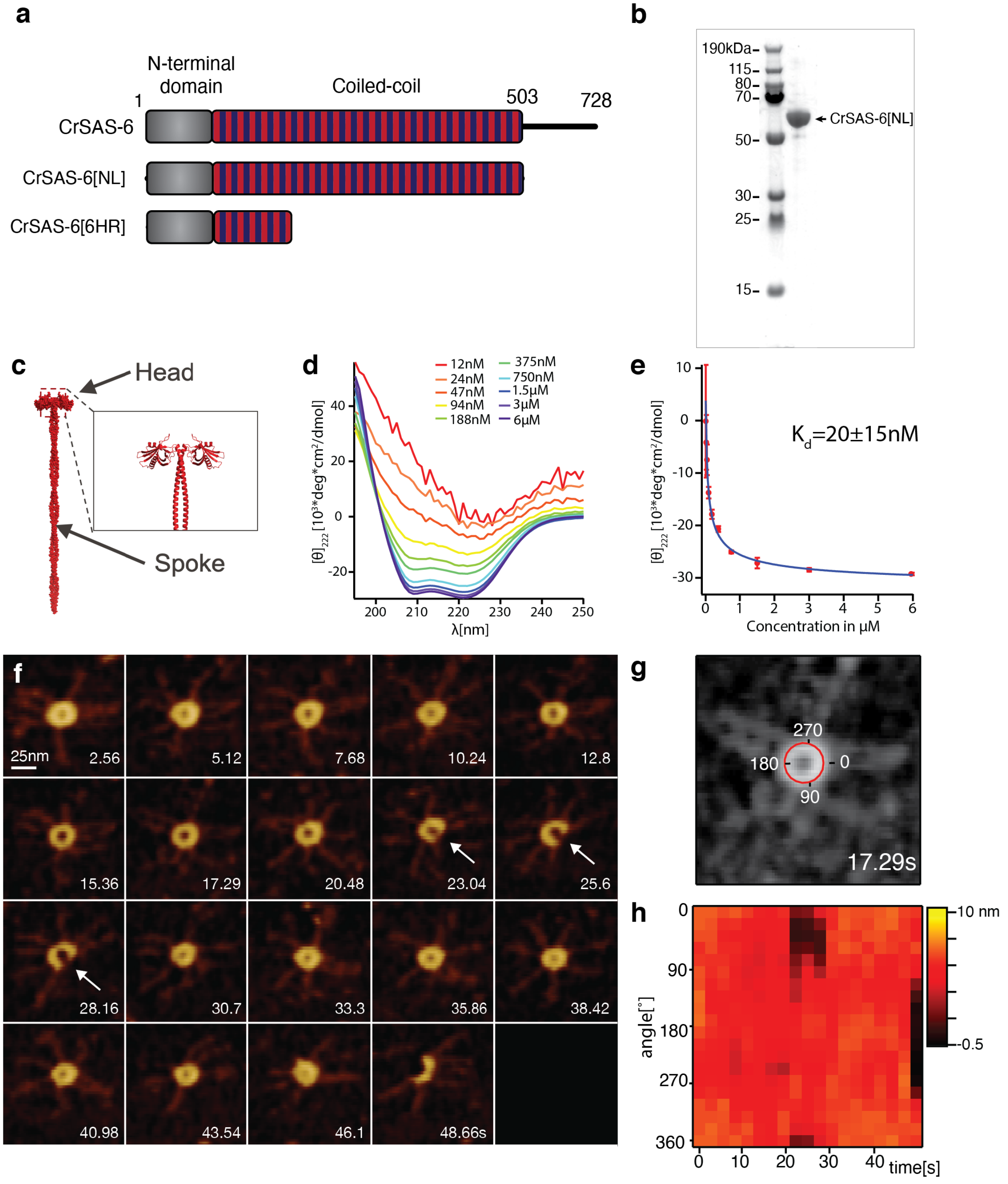

Supplementary Figure 1. CrSAS-6 proteins and ring opening analysis.

a Schematic of full length (728 amino acid long) CrSAS-6 protein (top), CrSAS-6[NL] protein used in the PORT-HS-AFM experiments (middle, amino acids 1-503), and CrSAS-6[6HR] protein used for the MD simulations (bottom, amino acids 1-226).

b Comassie staining of SDS-PAGE with recombinantly expressed and purified Cr-SAS-[NL], with an indication of molecular weight markers in kDa.

c Model of the CrSAS-6[NL] structure generated by combining the crystal structure of the CrSAS6[6HR] (PDBID:3Q0X, inset) with the rest of the coiled-coil generated *in silico* (see Modeling of SAS-6[NL] section in Methods).
d Circular dichroism (CD) spectra of CrSAS-6[NL] at indicated protein concentrations.
e Fit (blue line) of the CD signal at 222 nm as a function of concentration (red circles average of N=5 repeats, error bars represent the standard deviation) with a two-state model, yielding a dissociation constant of ~20±15 nM. Note that the high noise at concentration below 50 nM is reflected in the corresponding high standard deviation.
f Imaging of SAS-6 ring with PORT-HS-AFM, from full ring to complete disruption (time indicated in seconds; 2.56 s frame rate). Open rings (marked by white arrows) are observed at time-points 23.04, 25.6 and 28.16.
g Circular profile (red line) fitted to the ring formed by the head domains; numbers indicate angles in degrees.
h Corresponding heights along the circular profile over time. Regions with lower height (here: between time-points 23.04 and 28.16) in plot profiles were used to assign frames with potential ring opening events, which were then manually inspected to verity *bona fide* opening and closing transitions.

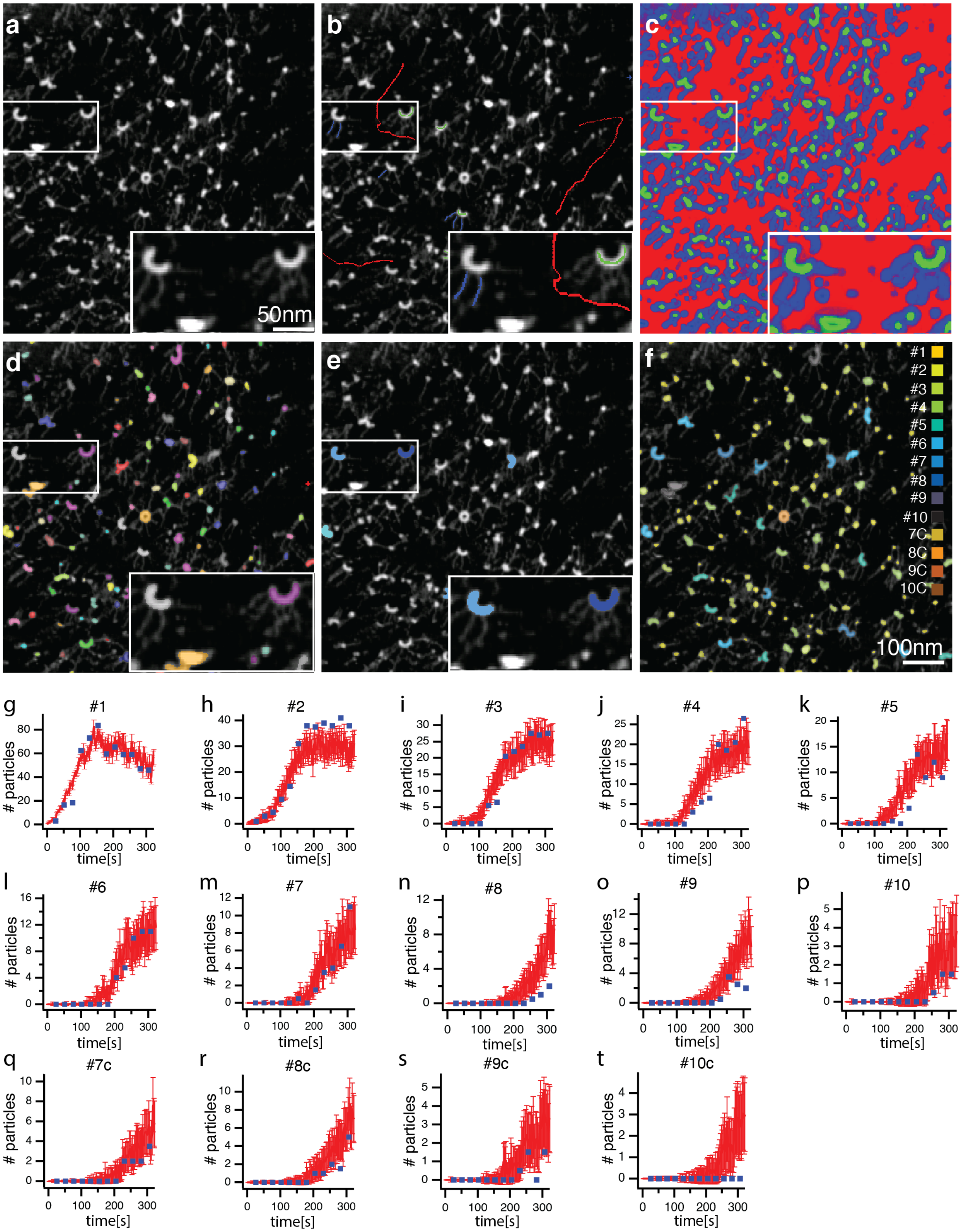

a
b
c
d
e
f
50nm
100nm
#1
#2
#3
#4
#5
#6
#7
#8
#9
#10
7C
8C
9C
10C
g
h
i
j
k
l
m
n
o
p
q
r
s
t
#7c
#8c
#9c
#10c
particles
time[s]

Supplementary Figure 2. Segmentation and classification pipeline.

a Single frame from SAS-6 PORT-HS-AFM imaging. High magnification inset is shown on the bottom right for this and other panels in this figure.
b Different brushes drawn to identify background (red), head domains (green) and spokes (blue).
c Pixel prediction map representing background (red), head domains (green) and spokes (blue).
d Head domains within SAS-6 oligomers identified after thresholding. Colors are assigned randomly to each distinct oligomeric assembly at this stage.
e Manual classification of selected objects into one of 14-classes (1-10 open oligomers, plus 7- to 10- fold closed rings). Some oligomers with clearly identifiable spoke numbers are assigned to a specific class at this step (e.g. colored oligomers in the frame).
f Final classification based on the probability of belonging to a certain class for each detected object. The class with the highest probability is displayed in the corresponding color.
g-t Automatic (red, error bar indicates SEM) versus manual (blue squares, average of independent scoring between N=2 authors) classification of particles not used for training. More than 95% of the data points from manual classification are within the expected error (2 sigma from the automatic classification) for each type of oligomeric species.

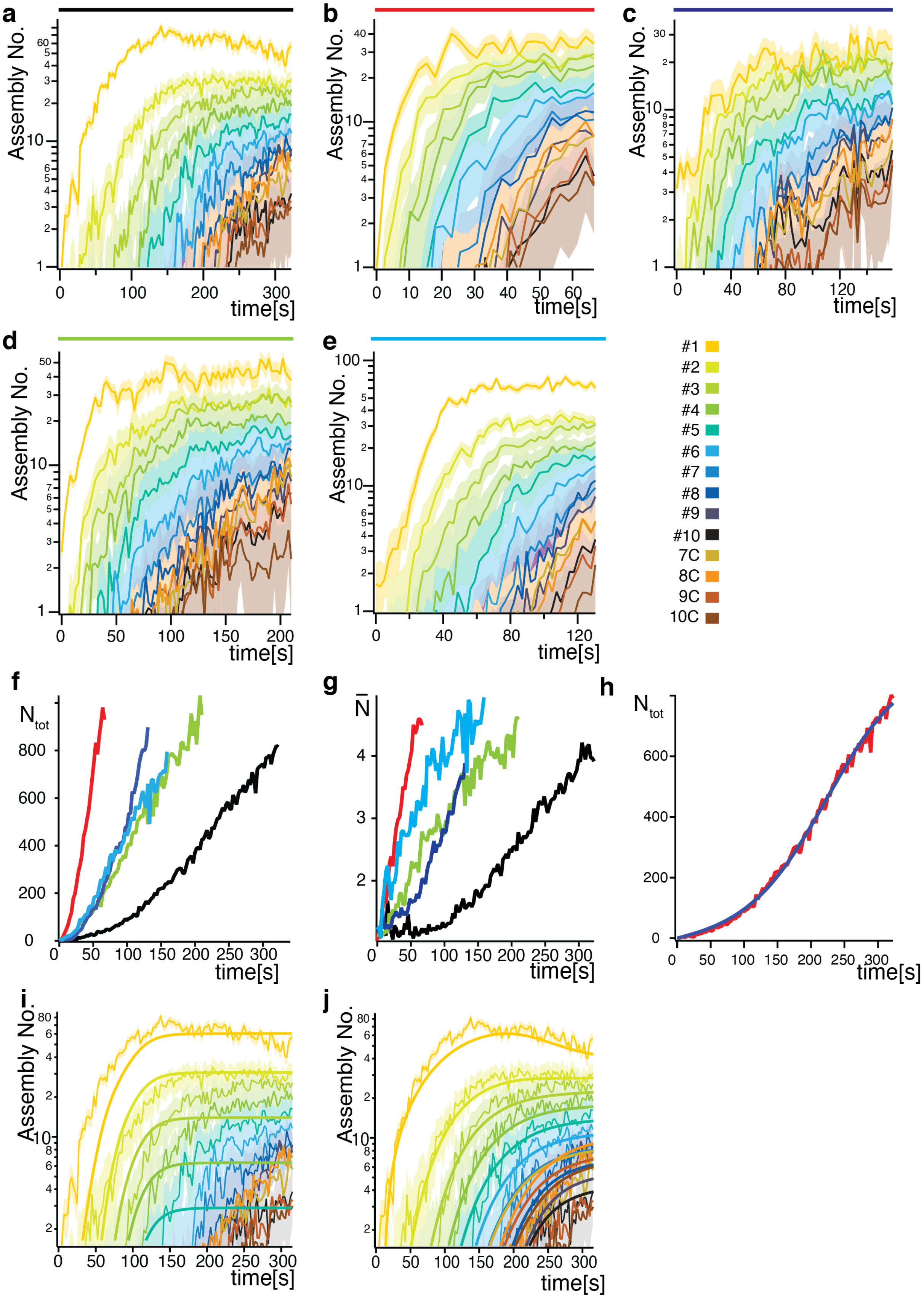

a
b
c
d
e
f
g
h
i
j
Assembly No.
time[s]
#1
#2
#3
#4
#5
#6
#7
#8
#9
#10
7C
8C
9C
10C
N_{tot}
$\bar{N}$

Supplementary Figure 3. SAS-6 assembly kinetics.

a-e SAS-6 assembly kinetics determined from the segmentation output of the five PORT-HS-AFM experiments analyzed in this study; panel a is the same as Fig. 3b. Colors above the plot correspond to lines shown in f-h. Error bars have been calculated as described in the section Kinetics Fitting Procedure in Methods.

f Total number of homodimers regardless of oligomeric state (N_{tot}) on the mica surface over time for the five PORT-HS-AFM experiments, each represented with a different color, as in a-e. The diversity in the traces reflects variable rates of homodimer adsorption on the surface, likely arising from variability in the pace of protein injection and landing on the mica.

g Average oligomerization degree $\bar{\boldsymbol{N}}$ over time for the five PORT-HS-AFM experiments. The variation in adsorption kinetic is reflected in the variable degree of polymerization over time.

h Total number of adsorbed homodimers on the mica surface in a PORT-HS-AFM experiment (black curve in f, corresponding to experiment shown in Fig. 3c and Supplementary Fig. 3a) regardless of oligomeric state (N_{tot}) as a function of time (red). The fitted curve (blue) was used to calculate the average concentration of dimers across the experiment to be $\hat{N} = 470$dimers/μm^2.

i Fit of the kinetics for the experiment shown in Fig. 3b and Supplementary Fig. 3a using the simpler Becker-Döring model considering only association and dissociation events of individual homodimers (see Model Comparison section in Methods).

j Fit of the kinetics for the experiment shown in Fig. 3b and Supplementary Fig. 3a using a model with 9 additional free parameters, in which all k_{on} and k_{off} values are identical except for those involving encounters with homodimers (see Model Comparison section in Methods).

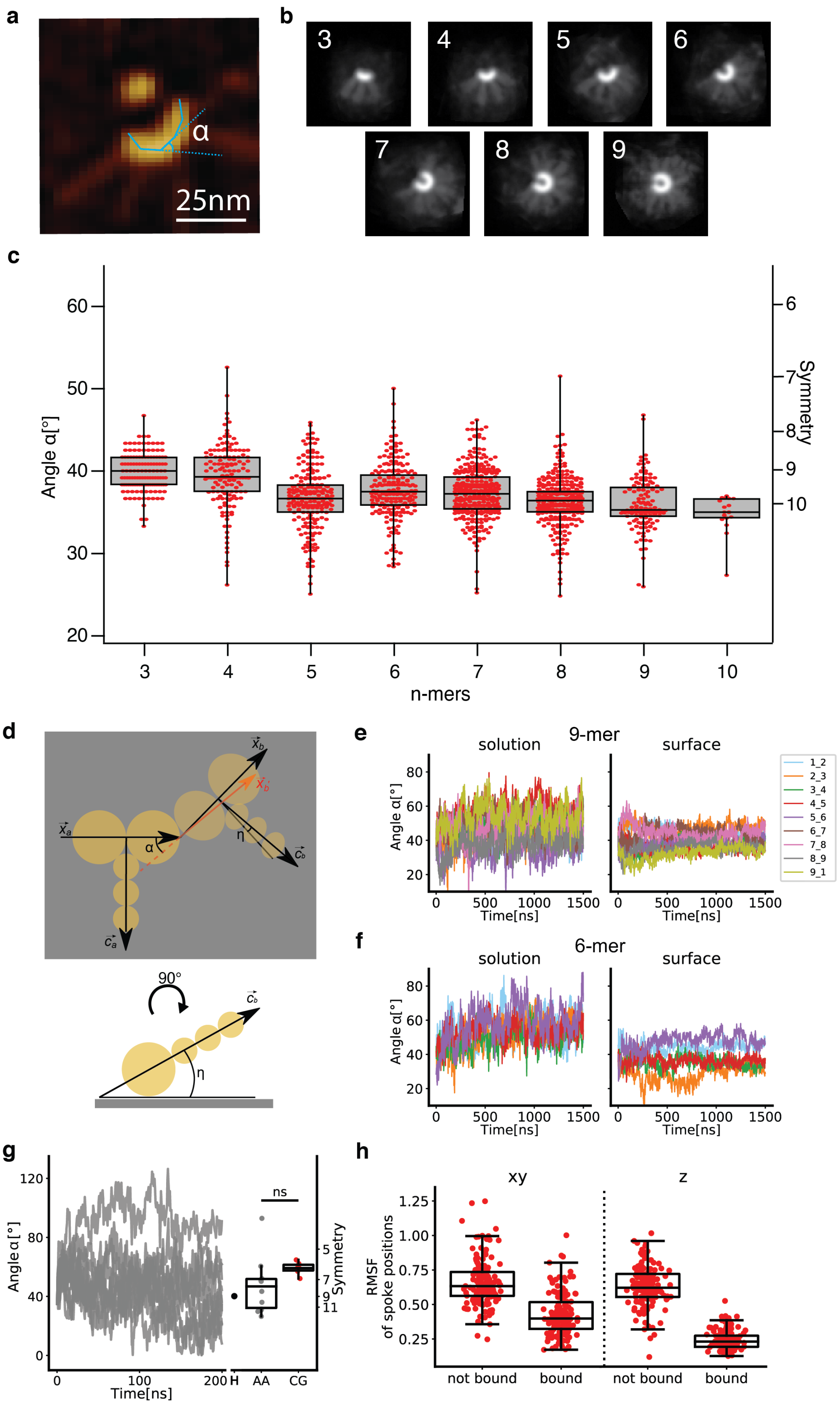

a
α
25nm
b
3
4
5
6
7
8
9
c
Angle α[°]
Symmetry
n-mers
d
90°
η
α
e
9-mer
solution
surface
Angle α[°]
Time[ns]
1_2
2_3
3_4
4_5
5_6
6_7
7_8
8_9
9_1
f
6-mer
solution
surface
Angle α[°]
Time[ns]
g
Angle α [°]
Time[ns]
ns
Symmetry
H
AA
CG
h
xy
z
RMSF
of spoke positions
not bound
bound
not bound
bound

Supplementary Figure 4. Angle determination between SAS-6 homodimers in PORT-HS-AFM experiments and in molecular dynamics (MD) simulations.

a Scheme of poly-line fit performed on PORT-HS-AFM data, with α_i being the fitted in-plane angle between SAS-6 homodimers.

b Average images of re-aligned open oligomeric species from 3-mers to 9-mers, based on the poly-line fit in the PORT-HS-AFM experiments (note that the number of 10-mers was too low to generate a meaningful average configuration).

c Box plot of α_i angles and predicted symmetries retrieved from fitting. The box shows the upper and lower limits as calculated by the Tukey method and mean, the whiskers extend from the minimum to the maximum of the experimental data.

d Schematic of measured angles between SAS-6 homodimers in the MD simulations; α: in-plane angle; η: torsion angle with respect to the surface. $\vec{\boldsymbol{x}}_{\boldsymbol{a}}$ and $\vec{\boldsymbol{x}}_{\boldsymbol{b}}$ are the vectors defining the orientations of the head domains of the two homodimers (designated as a and b), $\vec{\boldsymbol{c}}_{\boldsymbol{a}}$ and $\vec{\boldsymbol{c}}_{\boldsymbol{b}}$ are the vectors describing the orientation of the 6 heptad repeats and $\overrightarrow{\boldsymbol{x}'}_{\boldsymbol{b}}$ is the projection of $\vec{x}_b$ on the plane defined by $\vec{\boldsymbol{c}}_{\boldsymbol{b}}$ and $\vec{\boldsymbol{x}}_{\boldsymbol{b}}$.

e, f Individual time traces of in-plane angles α for 9-mer (e) and 6-mer (f) in the MD simulations, in solution and on a surface, as indicated. The different colored lines represent the time evolution of individual angles.

g Time traces (grey plots) and averages (grey scatters) of in-plane angles α in all-atom (AA) MD simulations of SAS-6[6HR] dimers, revealing the large range of explored angles and, therefore, the necessity to perform extensive sampling (left). Average angles measured in coarse-grain (CG, red scatters) MD simulations of SAS-6[6HR] dimers, as well as in a simulation of an N-terminal head domain dimer (black scatter, H, from [1]), which both fall within this range. ns: not significant, (independent two-sided t-test, p=0.38) (right). The box shows the upper (75%) and lower (25%) quartiles and median, the whiskers represent upper quartile+1.5*IQR (inter quartile range) and lower quartile-1.5*IQR, respectively.

h In-plane (xy) and off-plane (z) mobilities of spokes bound or not bound to the surface in the MD simulations. The box shows the upper (75%) and lower (25%) quartiles and median, the whiskers represent upper quartile+1.5*IQR (inter quartile range) and lower quartile-1.5*IQR, respectively

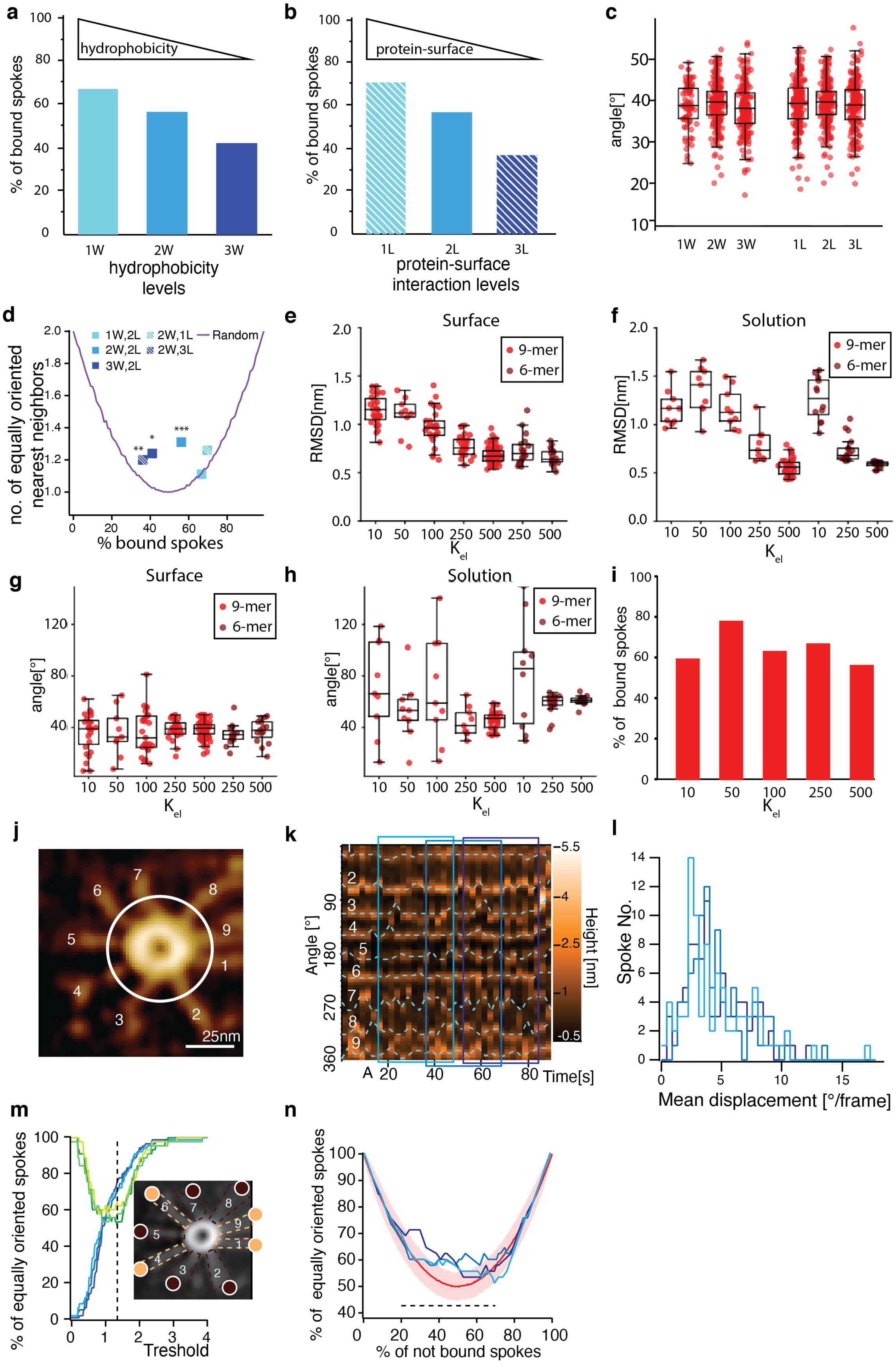

a
hydrophobicity
% of bound spokes
1W
2W
3W
hydrophobicity levels
b
protein-surface
% of bound spokes
1L
2L
3L
protein-surface interaction levels
c
angle[°]
1W 2W 3W 1L 2L 3L
d
1W,2L
2W,1L
Random
2W,2L
2W,3L
3W,2L
no. of equally oriented nearest neighbors
% bound spokes
e
Surface
9-mer
6-mer
RMSD[nm]
K_{el}
f
Solution
9-mer
6-mer
RMSD[nm]
K_{el}
g
Surface
9-mer
6-mer
angle[°]
K_{el}
h
Solution
9-mer
6-mer
angle[°]
K_{el}
i
% of bound spokes
K_{el}
j
25nm
k
Angle [°]
Height [nm]
Time[s]
l
Spoke No.
Mean displacement [°/frame]
m
% of equally oriented spokes
Treshold
n
% of equally oriented spokes
% of not bound spokes

Supplementary Figure 5. Analysis of spoke mobility in PORT-HS-AFM data set.
a Fraction of bound spokes at fixed protein-surface interaction (2L) and at varying surface-water interaction strength (1W, 2W, 3W) according to the Martini interactions table (where 1W is most hydrophobic and 3W most hydrophilic).
b Fraction of bound spokes at fixed surface-water interaction strength (2W) and at varying protein-surface interaction strength (1L, 2L, 3L) compared to the original graphene parametrization (where 1L is the strongest interaction and 3L the weakest).
c Box plots of plane angles in closed rings at different surface-water (W) and surface-protein (L) interactions (when W is varied L is fixed L=2, when L is varied W is fixed at W=2). The box shows the upper (75%) and lower (25%) quartiles and median, the whiskers represent upper quartile+1.5*IQR (inter quartile range) and lower quartile-1.5*IQR, respectively.
(same conventions are adopted also in e, f, g, h). Note that the angle range does not vary with the different surface parametrizations.
d Number of equally oriented nearest neighbors as a function of the fraction of bound spokes for different surface parametrizations (colored squares, as in **a** and **b**). The number of equally oriented nearest neighbors expected from a random distribution is shown by the continuous violet line. Asterisks indicate statistical significance (p values: $p_{2W3L}=0.0012$, $p_{3W2L}=0.038$, $p_{2W2L}<0.001$)
e, f RMSD values for individual homodimers at elastic constants of K_{el} = [10, 50, 100, 250, 500] kJ mol^{-1} nm^{-2} on the surface (**e**) and in solution (**f**). Note that most 6-mer simulations with K_{el} < 250 kJ mol^{-1} nm^{-2} were not stable.
g, h In plane angles at different elastic constants of K_{el} = [10, 50, 100, 250, 500] kJ mol^{-1} nm^{-2} on the surface (**g**) and in solution (**h**).
i The percentage of spokes bound to the surface for closed rings does not vary substantially as a function of the elastic constant.
j PORT-HS-AFM frame illustrating circle utilized to construct kymograph shown in k.
k Kymograph of spoke angle position and corresponding traces over time (blue dashed lines). The three examples for mean displacement (in l) were calculated considering only 13-frame blocks. Three examples of such randomly selected blocks are shown as rectangles with different hues of blue.
l Distribution of spoke mobility (lines in three different shades of blue) from ten closed 9-mer rings monitored by PORT-HS-AFM from three randomly selected 13-frame blocks.
m Three experimentally determined fractions of immobile spokes (green lines) at increasing thresholds of spoke mobility set to discriminate immobile versus mobile spokes. The three cases differ only for the random choice of the time blocks over which the average displacement is calculated. The corresponding fraction of spokes oriented in the same manner, either immobile or mobile, are also displayed (three blue lines). Inset shows an average image from a single 13-frame block with spokes classified as immobile (beige dashed lines and disks) or mobile (brown dashed lines and disks) at the selected threshold (black dashed vertical line).
n Fraction of spokes with similar mobility as a function of the fraction of mobile spokes expected from a random distribution determined from a Monte-Carlo simulation (red line, with standard deviation), and from the experimentally determined distributions (three blue lines) as in m. The variable fraction of mobile spokes (X axis) arises from varying the mobility threshold (see panel m). On average 100 randomly selected distributions were found to be more than 2 standard deviation apart from the theoretical distribution for random orientation in the region between 20-70% (dashed black line) of not bound spokes.

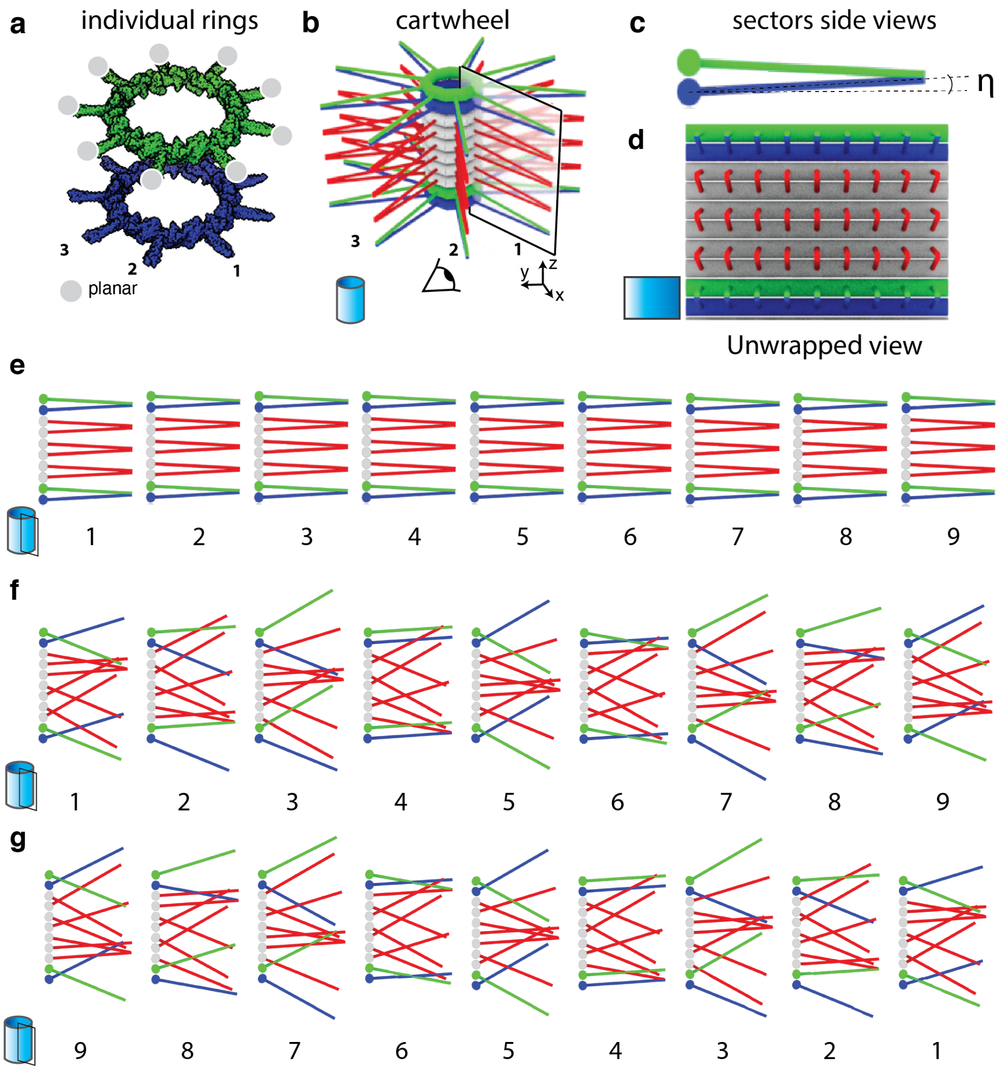

Supplementary Figure 6. Possible models of SAS-6 ring stacking.

a-d Current cartwheel paired model considering that SAS-6 spokes are near planar (grey discs) (**a**). Spokes from one ring point all in the same direction, leading to a regular 3D arrangement of spokes merging in pairs along each cartwheel sectors (**b**). Head domains and spokes of the first two and of the last two rings in the stack are represented in green and blue, respectively; for other SAS-6 molecules, head domains are colored in grey and spokes in red. The torsion angle η (**c**) is the same for each spoke within each ring as can be seen also from the lateral view unwrapped to obtain a complete assessment of the cartwheel surface (**d**).

e, f Individual side views of individual sectors (stacks of spokes) around the cartwheel circumference for the current paired model (**e**), and the proposed oscillating model (**f**).

g Reordered sectors of the oscillating model in anticlockwise direction. Comparing sectors placed on top of one another from panel (**f**) with those from panel (**g**) shows that the spoke organization in the two cases is distinct, thus exhibiting chirality.

Supplementary Table 1.

	7 closed (N=29)	8 closed (N=70)	9 closed (N=31)	10 closed (N=5)
**7 closed**	NA	<0.001	0.004	NS
**8 closed**	<0.001	NA	NS	NS
**9 closed**	0.004	NS	NA	NS
**10 closed**	NS	NS	NS	NA
	**7 open (N=18)**	**8 open (N=40)**	**9 open (N=23)**	**10 open (N=2)**
**7 open**	NA	0.02	0.004	NS
**8 open**	0.02	NA	0.014	NS
**9 open**	0.004	0.04	NA	NS
**10 open**	NA	NA	NS	NA

Significance difference (p-values) between mean open and closed lifetimes, comparing different symmetries as indicated (tested with F-ratio test, one-sided, on the mean values).

Supplementary Note 1: Coagulation-fragmentation equations for SAS-6 ring assembly

General remarks. Association and dissociation processes are ubiquitous in nature and can be described mathematically by the coagulation-fragmentation equations[2,3]. If association and dissociation only occur in steps of monomer addition or removal, these equations are known as the Becker-Döring equations[4,5]. Because these equations were originally conceived for processes that are dominated by association rather than dissociation and that can grow to very large sizes (e.g. growth of ice crystals, river deltas or planets), their mathematical treatment often focuses on the limit of large systems.

In the context of growth of supramolecular assemblies in biological systems, the dissociation process tends to be of the same nature as the association process; in particular, the binding and unbinding of proteins involves the association and dissociation of the same contacts in the binding interface. Moreover, system size tends to be finite and limited by some maximum. Predictions of the coagulation-fragmentation equations have been shown in the case of SAS-6 ring formation to agree with the results from Brownian dynamics computer simulation of patchy particles forming five- or nine-membered rings[6,7]. Because the transport process underlying association in such a patchy particle model is purely diffusive, it was possible to demonstrate that perfect agreement with the coagulation-fragmentation equations can be achieved by appropriately incorporating the diffusion process into the association rates of the coagulation-fragmentation equations, thereby combining spatial and kinetic modeling. In fact, the diffusion constants also entered in the dissociation rates due to the condition of detailed balance[6,7]. This earlier work now allows us to study the cases of diffusion-limited and reaction-limited regimes. In each case, one can identify the time scales for the growth dynamics and the steady states. In addition, one can study the role of absorbing boundaries, e.g. a stabilizing effect of ring closure that arises when a sufficiently large number of proteins have assembled.

Here we show that in the reaction-limited regime, the coagulation-fragmentation equations simplify to a form that effectively depends only on one unknown parameter, the ratio of bond association and dissociation rates, which can be extracted from experimental data and converted into an effective equilibrium constant. We also discuss the deviations that occur due to transport processes.

Coagulation-fragmentation equations. We first introduce the coagulation-fragmentation equations in their general form and then specify them for the purpose of the SAS-6 assembly process.

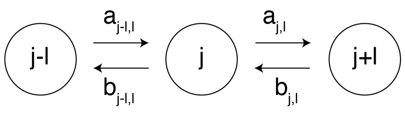

Supplementary Figure N1: Reaction scheme for the coagulation-fragmentation equations.

Association rates $a_{j,l}$ and dissociation rates $b_{j,l}$ are labeled by indices that denote which fragment sizes are involved. Note that in principle the rates could be very different dependent on fragment size because the reactions of differently sized fragments can involve very different transport and reaction processes. The special case $l = 1$ corresponds to a monomer addition and removal scheme, in which case the coagulation-fragmentation equations are called Becker-Döring equations.

We follow the nomenclature from[2]. The concentration of a fragment of size j is denoted by c_j. As shown in Supplementary Fig. N1, we have to consider two association and two dissociation processes that can change the concentration of a cluster of size (with $j \geq 2$). Using the law of mass action and summing over all possible sizes of the reaction partners, we obtain the following system of ordinary differential equations:

Equation 1

$$\dot{c}_j(t) = \frac{1}{2}\sum_{l=1}^{j-1} a_{j-l,l}c_{j-l}c_l - \sum_{l=1}^{N-j} a_{j,l}c_jc_l - \frac{1}{2}\sum_{l=1}^{j-1} b_{j-l,l}c_j + \sum_{l=1}^{N-j} b_{j,l}c_{j+l}$$

The signs in front of the four different terms denote if the concentration c_j increases or decreases by the corresponding process. Usually the coagulation-fragmentation equations are studied in the limit $N \to \infty$, but here we consider a maximal cluster size N.

The first term describes the coagulation of a $(j-l)$-cluster with a l-cluster into a j-cluster. The factor of $1/2$ reflects that the sum includes two terms that are equivalent, e.g. both a (1,4)- and a (4,1)-encounter can give a 5-cluster, but they represent the same process. Alternatively, one could have left out the factor of $1/2$ and restrict the summation to one of the two pairs[6,7]. The second term describes the coagulation of a j-cluster with a l-cluster into a $(j+l)$-cluster. In both cases, the association rate $a_{j,l}$ describes the probability with which a cluster of size j encounters and reacts with a cluster of size l. Obviously, we have $a_{j,l} = a_{l,j}$. Because of the product of the two concentrations resulting from mass action, these equations are non-linear. In 3D, the physical dimension of the association rates $a_{j,l}$ is $1/Ms$ or m^3/s, whereas it would be m^2/s in 2D. These dimensions reflect the fact that coagulation is bimolecular and depends on the concentration of a partner. In particular, this implies a transport process that brings a partner to close proximity before binding can occur.

The third term in Equation 1 describes how a j-cluster decays into a $(j-l)$-cluster and a l-cluster. Again, we have a factor of $1/2$ to avoid overcounting. The fourth term describes the decay of a $(j+l)$-cluster into a j-cluster and a l-cluster, thus increasing the number of j-clusters. For the dissociation rate, we also have $b_{j,l} = b_{l,j}$ irrespective of the exact mechanism of fragmentation. In both 3D and 2D, the physical dimension of the dissociation rates $b_{j,l}$ is $1/s$. This reflects the fact that fragmentation is a unimolecular reaction, starts from a situation of close encounter and does not depend on concentration of any partner.

We note that the case of two equally sized clusters requires extra care[6]. For association between n equally sized clusters, there are $n(n\text{-}1)/2$ possible pairings. Thus, in the thermodynamic limit of large system size, when the law of mass action becomes valid and when $n\text{-}1 \approx n$, this requires a factor of $1/2$, which is already contained in the notation of Equation 1.

For $j = 1$ and $j = N$, obviously two of the four processes are not possible and the corresponding terms will be missing. For these special cases, sometime the rates are redefined with a factor of 2 (e.g. $a_{1,1} = 2a_1$ for the Becker-Döring equations), to obtain a more symmetric definition of the system of equations in terms of fluxes[2].

One important consistency check of the coagulation-fragmentation equations from Equation 1 is that because they are formulated for a closed system here, they should obey mass conservation based on the symmetry of the matrices $a_{j,l}$ and $b_{j,l}$:

$$\sum_{j=1}^{N} j\,\dot{c}_j\,(t) = 0$$

The simplest test example would be N = 3, the formation of a trimer. Then only four reactions are possible: 1 + 1 → 2; 2 + 1 → 3; 3 → 2 + 1; 2 → 1 + 1. The system of ordinary differential equations following from Equation 1 reads:

$$\dot{c}_1(t) = -a_{1,1}c_1^2 - a_{1,2}c_1c_2 + b_{1,1}c_2 + b_{1,2}c_3$$

$$\dot{c}_2(t) = \frac{1}{2}a_{1,1}c_1^2 - a_{1,2}c_1c_2 - \frac{1}{2}b_{1,1}c_2 + b_{1,2}c_3$$

$$\dot{c}_3(t) = a_{1,2}c_1c_2 - b_{1,2}c_3$$

One sees that these equations are non-linear because the monomer concentration c_1 varies in time. Importantly, one also can verify that mass conservation is obeyed, $\sum_{j=1}^{3} j\,\dot{c}_j\,(t) = 0$, and identify the different terms that balance each other after multiplication with the monomer number j per cluster. For the case N=10 studied here, there are many more terms, thus we do not document them explicitly. Using computer algebra, one can verify that they obey mass conservation as expected.

Effect of transport. In the coagulation-fragmentation equations from Equation 1, association is described by an association rate $\boldsymbol{a_{j,l}}$ that in general depends on the cluster sizes $\boldsymbol{j}$ and $\boldsymbol{l}$. These rates are determined by two aspects: first, a transport mechanism must bring the two clusters in close spatial proximity, and second, a reaction can then take place to generate the product. Here we consider diffusion to be the relevant transport process. To determine the relative importance of diffusion versus reaction, we write this sequence of events in the following scheme[6] (for simplicity, we drop the indices and consider $\boldsymbol{j}$ and $\boldsymbol{l}$ to be fixed):

$$A + B \underset{b}{\overset{a}{\rightleftharpoons}} C\,,\, A + B \underset{k_{Diff,b}}{\overset{k_{Diff,a}}{\rightleftharpoons}} AB \underset{k_d}{\overset{k_a}{\rightleftharpoons}} C$$

Here, A and B are two oligomers coagulating to a new oligomer C. Conversely, the oligomer C can fragment into oligomers A and B. The overall association rate a and the overall dissociation rate b are the ones that should enter the coagulation-fragmentation equations. $k_{Diff,a}$ and $k_{Diff,b}$ denote the diffusive association and dissociation rates, respectively, which have dimensions of m^3/s (m^2/s in two dimensions) and $1/s$ (independent of dimension), respectively. AB is the encounter complex separating the transport step from the reaction step. k_a and k_d are the association and dissociation rates, respectively, for the reaction from encounter of the complex to the final product; both have dimension $1/s$. These four rates can be identified in computer simulations of the patchy particle model.

For sufficiently good statistics, we will assume the encounter complex AB to be in steady state. From this we obtain the effective association and dissociation rates[8,9]:

Equation 2

$$\boldsymbol{a} = \frac{\boldsymbol{k_{Diff,a} k_a}}{\boldsymbol{k_a + k_{Diff,b}}},\ \boldsymbol{b} = \frac{\boldsymbol{k_{Diff,b} k_d}}{\boldsymbol{k_a + k_{Diff,b}}}$$

This in turn allows us to identify the dissociation constant, which is also the concentration at half-occupancy:

Equation 3

$$K_d = \frac{b}{a} = \frac{k_{Diff,b} k_d}{k_{Diff,a} k_a} = \frac{k_d}{V^* k_a}$$

Here we have identified the reactive volume $V^* = k_{Diff,a}/k_{Diff,b}$ as the physical factor which is required to convert the non-spatial rate k_a for bond formation into an association constant that reflects the concentration dependance. For simple cases, the reactive volume can be calculated analytically; for more complex cases, it has to be determined through computer simulations. For proteins like SAS-6, the typical value is set by the size of the contact region and therefore has a value below nm^3. For the five- and nine-ring configurations, the reactive volumes have been tabulated for all possible cluster combinations (j, l)[6,7].

Equation 2 allows us to identify the two regimes of reaction and diffusion control. In the reaction-limited regime ($k_a \ll k_{Diff,b}$), we get $a = V^* k_a$ and $b = k_d$. We further specify the situation of patchy particles, which have two opposing patches that lead to oligomerization, as is the case for the SAS-6 homodimers. Then the reaction volume is twice the reactive volume of one patch, $V^* = 2V_p^*$, and the microscopic association rate k_a is the rate of bond formation when two patches encounter each other in space. Thus $a = 2V_p^* k_a$. The microscopic dissociation rate can be identified with twice the internal rate of bond dissociation, $b = 2k_d$, because there are two ways to cut a $(j + l)$-cluster into a j-cluster and a l-cluster. For equally sized clusters, there is only one way to cut, but then the double number of clusters is produced, thus a factor of 2 is justified in this case as well. Now the coagulation-fragmentation equations form Equation 1 simplify to:

Equation 4

$$\dot{\boldsymbol{c}}_{\boldsymbol{j}}(\boldsymbol{t}) = \boldsymbol{V_p^* k_a} \sum_{l=1}^{j-1} \boldsymbol{c_{j-l}\, c_l} - \boldsymbol{2V_p^* k_a c_j} \sum_{l=1}^{N-j} \boldsymbol{c_l} - \boldsymbol{k_d (j-1) c_j} + \boldsymbol{2k_d} \sum_{l=1}^{N-j} \boldsymbol{c_{j+l}}$$

This is exactly the form of the coagulation-fragmentation equations used in the main text if one identifies $k_{on} = V_p^* k_a$ and $k_{off} = k_d$. Because for the patchy particles we also have investigated previously the role of diffusive transport[7], we now can address the effect of assuming a reaction-limited process.

Formation of a 9-fold SAS-6 ring without closure. We next discuss the assembly of a 9-fold ring without ring closure, not considering other ring symmetries. In contrast to the experimental situation, but in agreement with the usual situation studied with the coagulation-fragmentation equations, we consider that we start with a certain number of monomers, which then are continuously used up. Using molecular information and affinity measurements from the literature, this system has been parametrized and compared to patchy particle simulations previously[7]. Because assembly in solution at the centrosomal concentration of c = 5 μM (corresponding to e.g. 125 particles in a 346 nm^3 box) was found in computer simulations to be very slow (time scale of days), a parametrization with enhanced reactivity but with the same known dissociation constant has been developed, which we use here as a reference case. In this parametrization, we have $V^* = 0.0585\ nm^3$, $k_a = 4$ / ns and $k_d = 1.06\ 10^{-6}$ / ns. Using equation 3, this leads to $K_d = 7.5$ μM for the patchy particles, which in turn corresponds to the experimentally measured $K_d = 60$ μM for single N-term domains (factor 8 due to combinatorics of patches). The diffusive on-rate has been simulated to be $k_{Diff,a} = 3\ 10^5$ / M s for the SAS-6 homodimers, which is three orders of magnitude smaller than the Smoluchowski rate for isotropic proteins and suggests that assembly of SAS-6 in solution is diffusion-limited in this case[10]. Indeed, the corresponding diffusive off-rate of our parametrization is $k_{Diff,b} = k_{Diff,a} / V^* = 8.55\ 10^{-3}$ / ns, which is much smaller than k_a. To explore the reaction-controlled regime, one has to lower both k_a and k_d by three orders of magnitude, so that k_a becomes smaller than $k_{Diff,b}$ and $K_d = k_d / (V^* k_a)$ stays constant at the experimentally known value.

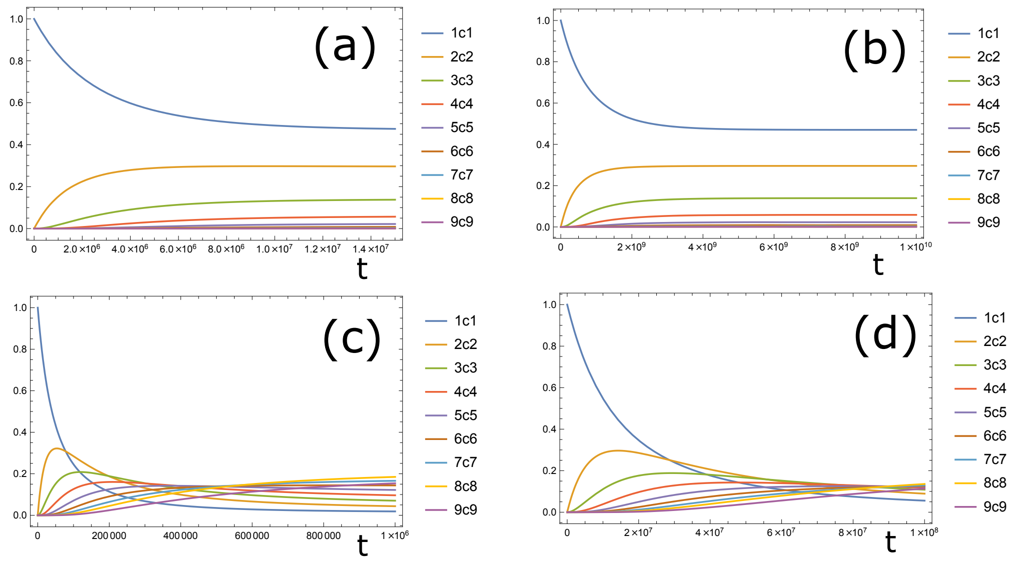

Supplementary Figure N2: Formation of a 9-fold SAS-6 ring using the coagulation-fragmentation equations taking into account diffusive transport and with the parametrization of enhanced reactivity from [7].
Time t measured in ns, concentrations c_j normalized by c_1. **a** *Reaction rates $k_a = 4$ / ns and $k_d = 1.06\ 10^{-6}$ / ns, concentration c = 5 μM, reactive volume for homodimers $V^* = 0.0585\ nm^3$, dissociation constant $K_d = 7.5$ μM, diffusive association rate $k_{Diff,a} = 3\ 10^5$ / Ms. At this concentration in solution, no rings and only small oligomers are formed in solution. The system is diffusion-limited.* **b** *When both k_a and k_d are reduced by three orders of magnitude, the system becomes reaction-limited and much slower (note the different scale on the time axis), but the dynamics is qualitatively very similar.* **c** *For strongly increased concentration c = 500 μM, rings are formed even in solution. Otherwise, parameters as in (a).* **d** *Again, going to reaction-control by decreasing both k_a and k_d by three orders of magnitude like in (b) does not change the dynamics qualitatively, except that the dynamics becomes much slower.*

In Supplementary Fig. N2, we plot the time evolution for these parameter values using the exact values for the reactive volumes and diffusive rates obtained in calibration simulations for the patchy particles and tabulated in[6]. Supplementary Fig. N2a corresponds to Figure S4 of[7] and demonstrates that in solution no rings are formed even at higher concentration (time measured in ns). This situation is strongly diffusion-controlled, but Supplementary Fig. N2b shows that reaction-control does not really change the picture, except for making the dynamics much slower. Formation of rings can be obtained only at high concentration, as shown in Supplementary Fig. N2c, where concentration has been increased by a factor of 100 compared to Fig. N2a. In Fig. N2d, we show that also in this case, reaction-control qualitatively gives very similar results, except for making assembly much slower again. We conclude that the assembly dynamics of SAS-6 is mainly determined by concentration and less by whether it is diffusion- or reaction-controlled.

Ring closure. We finally add the important aspect that after reaching the upper size limit $\boldsymbol{N}$, the ring can undergo a further step of closure. This step is characterized by a rate $\boldsymbol{k_a^{intra}}$ that does not depend on transport, but should be determined by the energy gained during ring closure. Because experimentally we have observed rings with sizes ranging between 7 and 10, we now set N=10 and allow for an additional (closed) state of all clusters with $\mathbf{7 \leq \boldsymbol{j} \leq 10}$. This leads to the final form of the equations:

$$
\begin{aligned}
\dot{c}_j(t) &= k_{on}\sum_{l=1}^{j-1} c_{j-l}\, c_l - 2k_{on}c_j\sum_{l=1}^{N-j} c_l + 2k_{off}\sum_{l=1}^{N-j} c_{j+l} - k_{off}(j-1)c_j \\
&\quad -k_{close}^{j}\sum_{l=7}^{10}\delta_{jl}\, c_j + k_{open}^{j}\sum_{l=7}^{10}\delta_{jl}\,\overline{c}_j \\
\dot{\overline{c}}_j(t) &= -k_{open}^{j}\overline{c}_j + k_{close}^{j}c_j
\end{aligned}
$$

The first equation is valid for $2 \leq j \leq 9$. For $j = 1$ and $j = N$, two of these terms are missing. The second equation is valid for $7 \leq j \leq 10$.

Supplemental References

1. Hilbert, M. *et al.* SAS-6 engineering reveals interdependence between cartwheel and microtubules in determining centriole architecture. *Nature Cell Biology* **18**, 393–403 (2016).
2. da Costa, F. P. Mathematical Aspects of Coagulation-Fragmentation Equations. in 83–162 (Springer, Cham, 2015). doi:10.1007/978-3-319-16121-1_5.
3. Wattis, J. A. D. An introduction to mathematical models of coagulation–fragmentation processes: A discrete deterministic mean-field approach. *Physica D: Nonlinear Phenomena* **222**, 1–20 (2006).
4. Ball, J. M., Carr, J. & Penrose, O. The Becker-Döring cluster equations: Basic properties and asymptotic behaviour of solutions. *Communications in Mathematical Physics* **104**, 657–692 (1986).
5. Becker, R. & Döring, W. Kinetische Behandlung der Keimbildung in übersättigten Dämpfen. *Annalen der Physik* **416**, 719–752 (1935).
6. Klein, H. C. R. & Schwarz, U. S. Studying protein assembly with reversible Brownian dynamics of patchy particles. *The Journal of Chemical Physics* **140**, 184112 (2014).
7. Klein, H. C. R., Guichard, P., Hamel, V., Gönczy, P. & Schwarz, U. S. Computational support for a scaffolding mechanism of centriole assembly. *Scientific Reports* **6**, 27075 (2016).
8. Eigen, M. Diffusion Control in Biochemical Reactions. in *Quantum Statistical Mechanics in the Natural Sciences* 37–61 (Springer US, 1974). doi:10.1007/978-1-4613-4532-9_3.
9. Shoup, D. & Szabo, A. Role of diffusion in ligand binding to macromolecules and cell-bound receptors. *Biophysical journal* **40**, 33–9 (1982).
10. Schreiber, G., Haran, G. & Zhou, H.-X. Fundamental Aspects of Protein−Protein Association Kinetics. *Chemical Reviews* **109**, 839–860 (2009).
