# Supplementary material for: Kinetic and structural roles for the surface in guiding SAS-6 self-assembly to direct centriole architecture
Source: Nat Commun. 2021 Oct 26;12:6180. doi: 10.1038/s41467-021-26329-1 (PMC8548535; doi:10.1038/s41467-021-26329-1)
Supplement: Supplementary file 3 — Description of Additional Supplementary Files [file 41467_2021_26329_MOESM3_ESM.pdf]

Description of Additional Supplementary Files

File Name: Supplementary Movie 1
Description: **SAS-6 oligomerization and ring assembly**
PORT-HS-AFM movie showing progressive adsorption of recombinantly expressed SAS-6 homodimers on the mica surface, followed by higherorder oligomerization and then ring formation. The color scale represents height and is the same as in Fig. 1. Time is in seconds from the beginning of the recording. Insets on the right show individual assembly/disassembly processes.

File Name: Supplementary Movie 2
Description: **SAS-6 ring opening and closing**
PORT-HS-AFM movie of a SAS-6 ring preformed on mica illustrating its opening, closing and eventual disassembly/desorption. The color scale represents height and is the same as in Fig. 1. Time is in seconds from the beginning of the recording.

File Name: Supplementary Movie 3
Description: **S-Shaped structure assembly**
PORT-HS-AFM movie of SAS-6 assembling into a transient S-Shaped conformation. The color scale represents height and is the same as in Fig. 1. Time is in seconds from the beginning of the recording.
