# Supplementary material for: Kinetic and structural roles for the surface in guiding SAS-6 self-assembly to direct centriole architecture
Source: Nat Commun. 2021 Oct 26;12:6180. doi: 10.1038/s41467-021-26329-1 (PMC8548535; doi:10.1038/s41467-021-26329-1)
Supplement: Supplementary file 7 — Reporting Summary [file 41467_2021_26329_MOESM7_ESM.pdf]

nature research

Corresponding author(s): Pierre Gonczy

Last updated by author(s): 20/09/2021

Reporting Summary

Nature Research wishes to improve the reproducibility of the work that we publish. This form provides structure for consistency and transparency in reporting. For further information on Nature Research policies, see our Editorial Policies and the Editorial Policy Checklist.

Statistics

For all statistical analyses, confirm that the following items are present in the figure legend, table legend, main text, or Methods section.

n/a	Confirmed	
☐	☒	The exact sample size (*n*) for each experimental group/condition, given as a discrete number and unit of measurement
☒	☐	A statement on whether measurements were taken from distinct samples or whether the same sample was measured repeatedly
☐	☒	The statistical test(s) used AND whether they are one- or two-sided<br>*Only common tests should be described solely by name; describe more complex techniques in the Methods section.*
☒	☐	A description of all covariates tested
☐	☒	A description of any assumptions or corrections, such as tests of normality and adjustment for multiple comparisons
☐	☒	A full description of the statistical parameters including central tendency (e.g. means) or other basic estimates (e.g. regression coefficient) AND variation (e.g. standard deviation) or associated estimates of uncertainty (e.g. confidence intervals)
☐	☒	For null hypothesis testing, the test statistic (e.g. *F*, *t*, *r*) with confidence intervals, effect sizes, degrees of freedom and *P* value noted<br>*Give P values as exact values whenever suitable.*
☒	☐	For Bayesian analysis, information on the choice of priors and Markov chain Monte Carlo settings
☒	☐	For hierarchical and complex designs, identification of the appropriate level for tests and full reporting of outcomes
☒	☐	Estimates of effect sizes (e.g. Cohen's *d*, Pearson's *r*), indicating how they were calculated

Our web collection on statistics for biologists contains articles on many of the points above.

Software and code

Policy information about availability of computer code

Data collection	Custom Labview Software (already described in Nievergelt, A. P., Banterle, N., Andany, S. H., Gönczy, P. & Fantner, G. E. High-speed photothermal off-resonance atomic force microscopy reveals assembly routes of centriolar scaffold protein SAS-6. Nature Nanotechnology 13, 696–701 (2018)), Gromacs (v. 2018.5), setup of simulation systems: UCSF Chimera (v.1.12), Swiss Model (https://swissmodel.expasy.org), Gromacs (2018), VMD (v. 1.9.3), martinize.py script (version 2.6, Marrink et al., 2007), JASCO (Spectra Manager 1 Software)
Data analysis	Gwyddion (v. 2.44), Ilastik (v.1.3.2), IgorPro (v. 8.04), Fiji (Imagej v.1.2.0), Gromacs (2018), Python3 (packages: numpy v.1.18.1, pandas v.1.0.1, scipy v.1.4.1, matplotlib v.3.1.3), Nanotube Builder, CCBuilder (2.0), Custom code (https://github.com/UPGON/HS_AFM_SAS_6_Analysis.git)

For manuscripts utilizing custom algorithms or software that are central to the research but not yet described in published literature, software must be made available to editors and reviewers. We strongly encourage code deposition in a community repository (e.g. GitHub). See the Nature Research guidelines for submitting code & software for further information.

Data

Policy information about availability of data

All manuscripts must include a data availability statement. This statement should provide the following information, where applicable:

- Accession codes, unique identifiers, or web links for publicly available datasets
- A list of figures that have associated raw data
- A description of any restrictions on data availability

The MD results are deposited and publicly available at the open-access research data repository of Heidelberg University (heiDATA (uni-heidelberg.de: https://doi.org/10.11588/data/3NKHAY). All the other data supporting the findings of this study are available from the authors on reasonable request; see author contributions for specific data sets.

Field-specific reporting

Please select the one below that is the best fit for your research. If you are not sure, read the appropriate sections before making your selection.

- [x] Life sciences
- [ ] Behavioural & social sciences
- [ ] Ecological, evolutionary & environmental sciences

For a reference copy of the document with all sections, see nature.com/documents/nr-reporting-summary-flat.pdf

Life sciences study design

All studies must disclose on these points even when the disclosure is negative.

Sample size	No statistical methods were used to predetermine the sample size.
Data exclusions	Several AFM movies were discarded based on image quality criteria (roughly 9 out of 10). Opening and closing events were also discarded if the symmetry of the corresponding ring was unclear.
Replication	AFM of dynamic assembly and of preformed rings were performed at least five times with similar results. All molecular dynamics experiments where repeated at least three times per condition.
Randomization	Randomization was not necessary as samples were not allocated to groups.
Blinding	No double blinding of the experiment was performed given that the nature of the experiment consisted in the injection and observation of a known sample and not on the comparison between different conditions.

Reporting for specific materials, systems and methods

We require information from authors about some types of materials, experimental systems and methods used in many studies. Here, indicate whether each material, system or method listed is relevant to your study. If you are not sure if a list item applies to your research, read the appropriate section before selecting a response.

Materials & experimental systems

n/a	Involved in the study	
☒	☐	Antibodies
☒	☐	Eukaryotic cell lines
☒	☐	Palaeontology and archaeology
☒	☐	Animals and other organisms
☒	☐	Human research participants
☒	☐	Clinical data
☒	☐	Dual use research of concern

Methods

n/a	Involved in the study	
☒	☐	ChIP-seq
☒	☐	Flow cytometry
☒	☐	MRI-based neuroimaging
